# Revival of Tigers: Long-Term Trends (2009–2022) in the Relative Abundance Index of Tigers, Prey, and Anthropogenic Disturbance in Parsa National Park, Nepal

**DOI:** 10.3390/ani15182697

**Published:** 2025-09-15

**Authors:** Amir Maharjan, Tek Maraseni, Armando Apan, Benjamin L. Allen

**Affiliations:** 1 Institute for Life Sciences and the Environment, University of Southern Queensland, Toowoomba, QLD 4350, Australia; tek.maraseni@unisq.edu.au (T.M.); armando.apan@unisq.edu.au (A.A.); 2 Ministry of Forest and Environment, Government of Nepal, Singha Durbar, Kathmandu 44600, Nepal; 3 Northwest Institute of Eco-Environment and Resources, Chinese Academy of Sciences, Lanzhou 730000, China; 4 Institute of Environmental Science and Meteorology, University of the Philippines Diliman, Quezon City 1101, Philippines; 5 Centre for African Conservation Ecology, Nelson Mandela University, Gqeberha 6034, South Africa; benallendingo@gmail.com

**Keywords:** forest management regimes, prey, Relative Abundance Index, tiger conservation

## Abstract

Tigers (*Panthera tigris*) are apex predators and vital indicators of a healthy terrestrial ecosystem. Their effective conservation demands long-term data on their populations, prey abundance, and anthropogenic disturbances from humans and domestic animals across different forest management regimes. In this study, we analyzed camera trap datasets (2009, 2013, 2018, and 2022) from Parsa National Park, its buffer zone, and adjoining national forests. Using the Relative Abundance Index (RAI), we quantified the abundance of tigers, prey, humans, and domestic animals. Our findings revealed a significant increase (χ^2^ = 9.6; df = 3; *p* < 0.05) in the RAI of tigers (from 2.65 in 2009 to 7.11 in 2022) and their prey, coupled with a decrease in anthropogenic disturbances in the national park. Meanwhile, no significant differences in the RAI of tigers and their prey were observed in the buffer zone and national forests. We also found an increasing trend of human disturbances (RAI from 351.44 in 2009 to 389.7 in 2022) in the national forests. However, the abundance of domestic animals showed a decreasing trend across all three forest management regimes. Our results suggest that a reduction in anthropogenic disturbances has a more notable positive impact on tigers’ abundance than on their prey. This study emphasizes the need for directed conservation policies to reduce anthropogenic disturbances in buffer zones and national forests, while also addressing local needs and securing their goodwill for sustainable tiger conservation.

## Simple Summary

Increasing tiger populations in some regions have led to a rise in human–tiger conflicts, particularly in human-dominated landscapes. This study analyzed long-term camera trap data (2009–2022) to understand population trends of tigers, their prey, and anthropogenic disturbances from humans and domestic animals across three forest management regimes: Parsa National Park, its buffer zone, and adjoining national forests. The findings showed that tiger and prey populations increased within the national park, while anthropogenic disturbances declined. In contrast, there were no major changes in tiger and prey numbers in the buffer zone and national forests, and human disturbances were found to be increased in the national forests. Interestingly, the presence of domestic animals showed a declining trend across all three areas. This study suggests that reducing human disturbances supports faster growth in tiger populations than their prey. Therefore, to protect both tigers and their prey, it is important to combine efforts to reduce human impact with measures to restore prey populations. This study also highlights the need for stronger conservation policies in buffer zones and national forests that balance tiger protection with the needs of local communities.

## 1. Introduction

Tigers (*Panthera tigris*) are categorized as ‘Endangered’ by the International Union for Conservation of Nature (IUCN) Red List [1] and listed in the Convention on International Trade of Endangered Species of Flora and Fauna (CITES) Appendix I [2], prohibiting their international trade. They have already lost 93% of their historical range worldwide, primarily due to habitat loss, prey depletion, and anthropogenic disturbances [1,3,4]. Tigers hold the apex position of the food chain in terrestrial ecosystems and are, thus, known as an important indicator species signifying healthy ecosystems [5,6,7,8,9]. Currently, there exist six subspecies of tigers, found only in 10 tiger range countries (TRCs) [1]. Among these subspecies, the Bengal tiger (*Panthera tigris tigris*) is the most abundant, with the highest number of ~3800 individuals, and its habitat is limited to areas in India, Nepal, Bangladesh, and Bhutan [1,10,11]. However, nearly 50% of tiger prey species are currently threatened, with over 80% showing population declines, primarily due to habitat loss, hunting, and human–wildlife conflicts [12,13]. Ongoing anthropogenic pressures continue to adversely affect both tigers and their prey, exacerbating conservation challenges and increasing the frequency of human–tiger conflicts [14,15,16]

Camera traps are widely used in wildlife ecology studies [17], allowing researchers to estimate population size, species richness, occupancy, and activity patterns [18,19,20]. Camera traps are especially useful for studying elusive animals such as tigers and their prey species [5,21]. For species without distinct markings, such as ungulates, trapping rates (e.g., photo/trap night) are commonly used to estimate relative abundance [22,23]. The Relative Abundance Index (RAI) is a simple and widely used method for assessing relative changes in species abundance, which is usually calculated as the number of independent photographs of a focal species per 100 trap nights [24]. It is especially useful in situations where directly estimating population size is difficult and expensive, and it is often applied to monitor changes over time or to compare relative abundance across different sites [25,26]. Although use of the RAI has certain limitations, such as imperfect and variable detection [27,28], several studies (e.g., those by Palmer et al. [25] and Mandujano et al. [26]) support its utility as a rapid assessment tool for tracking population trends of the same species over time and across space, particularly for herbivores lacking distinctive markings. The RAI derived from randomly placed camera traps often correlates with density estimates from Capture–Mark–Recapture (SECR) and distance sampling, supporting its reliability as a proxy for abundance and making it a suitable tool for monitoring population trends [22,23,29,30,31].

Several tiger-related studies using the RAI have primarily been conducted within protected areas [32,33,34,35], with only a few exceptions outside protected areas [36]. This limited focus is concerning, as habitats outside protected areas are becoming increasingly important for tigers and their prey due to ongoing degradation of habitats within protected areas caused by human activities [37,38]. Moreover, comparative RAI studies examining population trends of tigers and their prey both within and outside protected areas across a shared landscape remain largely underexplored.

To address this gap, we conducted a long-term comparative study using camera trap data collected in 2009, 2013, 2018, and 2022 to assess the RAIs of tigers, their prey species, and anthropogenic disturbances exerted by humans and domestic animals across three contiguous forest management regimes in Nepal, including Parsa National Park (PNP), its buffer zone, and adjacent national forests. We tested the following hypotheses: (1) there is a significant difference in the abundance of tigers and their prey species in the three forest management regimes over time, and (2) high levels of anthropogenic disturbances from humans and domestic animals negatively influence the abundance of tigers and their prey.

The study area lies in the easternmost part of the Terai Arc Landscape (TAL), a transboundary landscape recognized for conserving biodiversity, with PNP representing a strictly protected area restricted to public access. This protected area is considered a high-priority area for tiger conservation, with the population increasing from 4 to 41 tigers between 2009 and 2022 [39,40]. Although ecological carrying capacity is dynamic by nature, this rapid increase suggests that PNP may have reached saturation [41], prompting dispersal of tigers into the surrounding buffer zone and national forests. Unlike PNP, the buffer zone allows local people to use forest products, while national forests permit both local communities and the government to utilize and sell forest products [42]. Comparative understanding of how these distinct forest management regimes influence wildlife and anthropogenic disturbances is essential for developing effective conservation strategies that extend beyond protected areas.

To attain our objectives, we applied the RAI based on O’Brien et al.’s study [43], using systematic camera trap data collected over four survey periods spanning 14 years. This study introduces several important innovations in tiger conservation research. First, it delivers a unique long-term comparative assessment of tiger and prey abundance alongside anthropogenic disturbances across three contiguous forest management regimes, a national park, buffer zone, and national forests, offering critical insights into tiger ecology beyond strictly protected areas. Second, by combining extensive camera trap data with analyses of human and domestic animal disturbances, this study reveals differential impacts of anthropogenic pressures on tigers and their prey, emphasizing the importance of reducing human interference for apex predator recovery. Lastly, this study advances methodological understanding by evaluating the utility of the RAI in monitoring wildlife trends over time, supporting its application as an effective, low-cost monitoring tool in complex landscapes. The findings have significant implications for habitat management, policy development, and human–wildlife conflict mitigation, particularly in areas where tiger conservation must extend beyond protected areas into more complex landscapes.

## 2. Materials and Methods

We obtained permission from Nepal’s Ministry of Forest and Environment (Approval number 079/80 2183) for this study and obtained the data from the Department of National Parks and Wildlife Conservation, Nepal. We also attained the human research ethics clearance ETH2023-0344 (HREC) from the University of Southern Queensland, Australia.

### 2.1. Study Area

**Figure 1:**
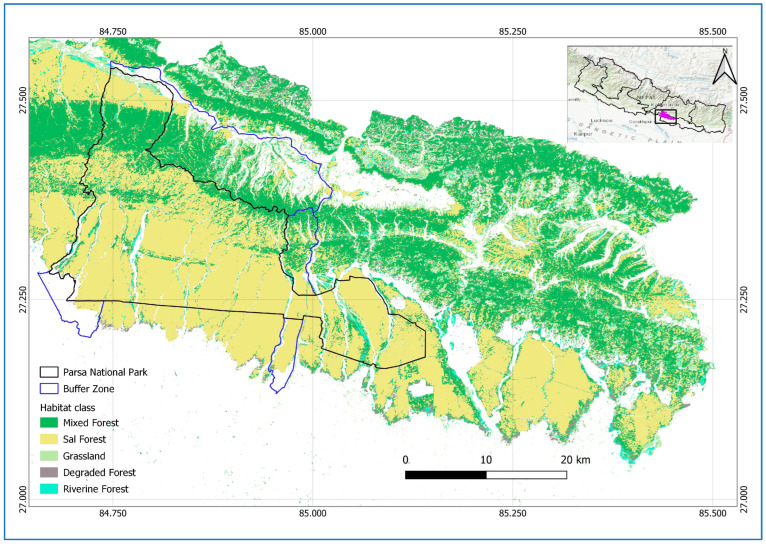
Location of study area: PNP (black solid line), its buffer zone (blue solid line), and national forests, Nepal. The forest area located to the south of PNP and its buffer zone boundaries, extending to the lower right side of the map, shows the national forests [45].

**Figure 2:**
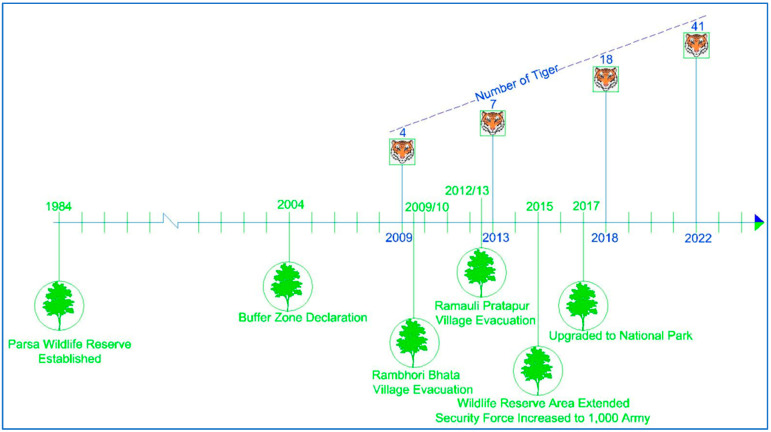
History of Parsa National Park with timeline of key events.

The study area encompasses 1758 km^2^ of Parsa National Park (PNP), its buffer zone, and national forests located in the Parsa, Bara, and Rautahat districts of Nepal (Figure 1). The entire area is a contiguous forest located in the easternmost part of the Terai Arc Landscape (TAL). The TAL is a transboundary region landscape, globally recognized for its commitment to conserving biodiversity and ecological integrity, while promoting the socio-economic well-being of local communities. The TAL is recognized as one of the world’s seven Restoration Flagships, highlighting its role as a leading model for sustainable landscape restoration by the United Nations [44]. Initially, PNP was designated as a wildlife reserve in 1984, comprising 499 km^2^ to protect wildlife such as tigers, but it was further extended by 127 km^2^ in 2015, and later upgraded to national park status in 2017. PNP is connected with Chitwan National Park, Nepal, to the west and the Valmiki Tiger Reserve, India, to the south. The 285.3 km^2^ buffer zone was declared in 2005, which surrounds PNP and includes forests, settlements, and agricultural land. The historical timeline, highlighting major events of PNP, is shown in Figure 2. Similarly, the national forests of the Parsa, Bara, and Rautahat districts, with a total area of 845.25 km^2^, are owned by the government, with the primary objective of forest utilization.

All of these forest areas comprise diverse wildlife, such as tigers (*Panthera tigris*), greater one-horned rhinoceros (*Rhinoceros unicornis*), leopards (*Panthera pardus*), Asian elephants (*Elephus maximus*), spotted deer (*Axis axis*), sambar deer (*Rusa unicolor*), wild boars (*Sus scrofa*), barking deer (*Muntiacus muntjac*), blue bulls (*Boselaphus tragocamelus*), king cobras (*Ophiophagus hannah*), pythons (*Python molurus*), giant hornbills (*Bucerous bicornis*), and many others. The floral diversity includes sal (*Shorea robusta*), *sisso* (*Dalbergia sissoo*), khayer (*Acacia catechu*), simal (*Bombax ceiba*), saj (*Terminalia tomentosa*), etc. A summary of all three forest management regimes is presented in Table 1.

In Nepal, national parks are strictly protected areas focused on conservation, where human activities are fully restricted to maintain biodiversity and ecosystems. Buffer zones, located around national parks, allow for regulated human use, supporting local communities through sustainable resource extraction, tourism, and development activities. These areas are designed to reduce human–wildlife conflicts while still benefiting surrounding populations [46,48,49]. National forests are managed by the government for multiple purposes, including timber production, grazing, and conservation. They have the fewest restrictions, offering resources to local communities while balancing ecological protection [42,48].

### 2.2. Camera Trapping

**Figure 3:**
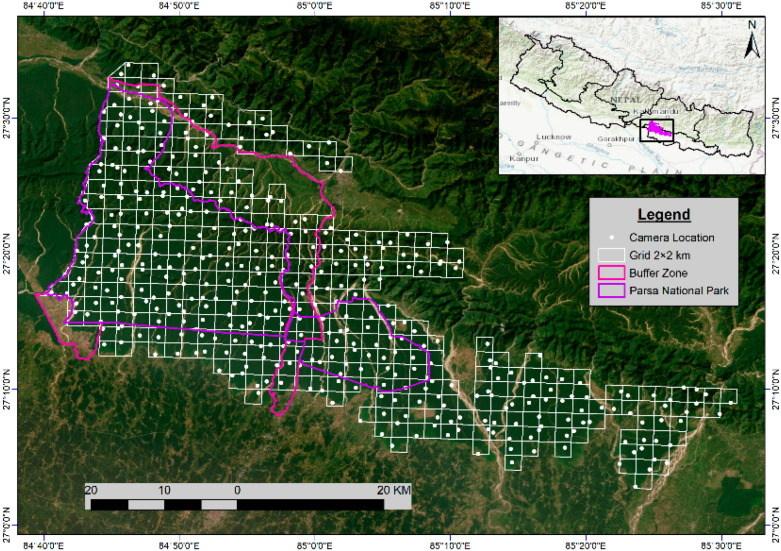
Location of study area showing PNP, its buffer zone, and national forests, along with the 2 × 2 sq. km grid layout with 364 GPS camera trap locations in the year 2022. The grid locations beyond the PNP and buffer zone are the national forest areas.

This study used camera trap data from Nepal’s national tiger surveys conducted in 2009, 2013, 2018, and 2022 (the latest), focusing on PNP, its buffer zone, and the adjoining national forests. The surveys were conducted each year during the winter–spring season (Dec–April) following the “Tiger and Prey Base Monitoring Protocol 2017” [50]. For the camera trap survey, the grid layout expanded from 117 (2 km × 2 km) in PNP in 2009 to 364 grids (Figure 3) up to the Rautahat district by 2022 (Table 2). In 2009, camera traps were placed only in PNP, but as evidence of tiger presence outside the national park increased, efforts were expanded in the buffer zone and adjoining national forests in subsequent years. The grids were established in the potential tiger habitats and consistently used for each census, with the exact location of camera trap station(s) on each grid chosen based on tiger signs such as pugmarks, scrapes, scat, water availability, and trails. At each station, a pair of cameras was mounted on trees or posts, at a height of 45 cm above the ground, facing each other at 6–8 m apart. The cameras were set to run for 24 h using white flash, and taking three pictures per trigger with no delay, for 16 days on average. Each camera and memory card was given a unique identification number for data recording and recognition, and the cameras were checked every two days to ensure continuous operation. The date, time, and camera ID were automatically printed on every image. Different types of cameras, including Reconyx (500 & 550), Bushnell Trophy Cam HD, Moultrie, Stealth, Cuddeback (C1), and Panthera (V5 &V6), were employed during this study. The position of each camera trapping station was systematically recorded using a handheld GPS device.

### 2.3. Data Collection

From the camera trap photographs collected, we extracted data on all tigers, prey species, humans, and domestic animals from PNP, its buffer zone, and national forests. We used the “Timelapse” program [53] to formulate data sheets and segregate photographs, recording each capture in an image with its corresponding location, date, time, and species, including the number of individuals observed. We considered the five major prey species of tigers, i.e., spotted deer (*Axis axis*), wild boar (*Sus scrofa*), sambar (*Rusa unicolor*), barking deer (*Muntiacus muntjak*), and gaur (*Bos gaurus*) [40,51,52]. Similarly, domestic animals (such as cows, buffaloes, goats, and dogs) and humans (including pedestrians, herders, fuelwood and timber collectors, poachers, and vehicles, including bicycles) were also recorded. To avoid pseudo-replication, we rated each photo as an independent capture if the time between consecutive photos of the same species was more than 30 min apart [43]. Photos with multiple individuals of the same species appearing in a single photo were considered as a single capture for that species. Multiple species appearing in a single photo were counted as separate captures for each species. Malfunctioned or damaged cameras, those with missing data, and cameras active for less than 3 days were excluded, and details of the camera traps considered for this study are presented in Supplementary Materials S1.

### 2.4. Data Analysis

Based on independent rates of photo capture of tigers, prey species, humans, and domestic animals, we calculated the Relative Abundance Index (RAI) for each category following the formula provided by O’Brien et al. [43].

$$\;RAI=\frac{A}{N}\times 100$$

where ‘*A*’ represents the total number of captures of a species by all cameras, and ‘*N*’ equals the total camera trap days during the study period.

We first calculated the station-wise RAI for each species, including anthropogenic disturbances, for each survey year. Subsequently, we computed the mean RAI along with its standard error (SE) for each species per year. This consistent approach was applied across all survey years, ensuring the statistical comparability of mean RAI values over time (see Table 3).

To test the first hypothesis, we performed the Friedman Rank-Sum Test to compare the RAI values of tigers and all five prey species across all four survey years. This was performed using the ‘friedman.test’ function in R version 4.3.1 in the interface RStudio. Similarly, for the post hoc test, we applied the Wilcoxon Signed-Rank Exact Test using the ‘wilcox.test’ function in R.

We treated anthropogenic disturbances (humans plus domestic animals) separately because changes in their abundance have an opposite relationship with wild animals [54,55]. We combined the RAIs of humans and domestic animals as anthropogenic disturbances, and the RAIs of all five prey species as prey, for the analysis of the line graph. To elucidate species relationships, we calculated the Spearman correlation coefficient (ρ) between the RAIs of tigers, prey species, humans, and domestic animals. Correlation coefficients between 0.5 and 0.7 indicated moderate relationships, where >0.7 indicated strong relationships [56]. The direction of the relationship (positive or negative) was determined by the sign of the coefficient. These graphs and correlations were used for testing the second hypothesis.

Similarly, all other statistical analyses, such as the mean, standard deviation, and range, were also carried out in R. MS Excel was used for creating pie diagrams, graphs, and boxplots, while Arc GIS 10.2 was employed for mapping purposes.

## 3. Results

We analyzed 716,968 camera trap photos from four surveys, extracting 34,161 independent captures of tigers, prey, humans, and domestic animals across 819 camera trap stations.

### 3.1. Percentage of Camera Trap Photographs and Independent Photo Captures

In PNP, the camera trap photographs captured the lowest percentage of tigers in 2013 (0.001%) and the highest percentage in 2022 (4%), with independent photo captures of n = 11 and n = 201 in the respective years. Among the prey species, barking deer and wild boar showed the highest percentage of photographs over time. Meanwhile, gaur had the lowest percentage of photographs, consistently at 1% every year, but independent captures increased steadily from 2009 (n = 4) to 2022 (n = 37). The percentage of photographs of humans fluctuated over time, but followed a declining trend in independent captures, with a minimum in 2022 (n = 449). Similarly, the photograph percentage of domestic animals was found to be fluctuating, while independent captures were found to be declining over time, with the highest number in 2013 (n = 1131) and the lowest in 2022 (n = 63).

In the buffer zone forest, tigers were recorded only in 2022, with independent captures of n = 38. Among the prey species, barking deer was found to be the highest in the percentage of photographs and independent captures. Meanwhile, gaur was found to be the lowest in percentage with independent captures from none to n = 16 in 2022. For humans, the percentage of photographs fluctuated over time from 2013 (58%) to 2022 (54%), while independent captures clearly showed an increasing trend from n = 705 to n = 3376 in the following years. Similarly, domestic animals also showed a steady increase, from 2018 (15%) to 2022 (43%), with independent captures of n = 413 and n = 1054, respectively.

In national forests, tigers were not recorded until 2013, while they started to be photographed in 2018 (1%) to 2022 (0.0014%), with independent captures of n = 44 to n = 40, respectively. Among the prey species, wild boar and barking deer were found to have the highest number of photographs, with wild boar having the highest independent captures over time. Gaur was sighted only from 2018, with independent captures of n = 43 in the following year. Human presence showed a drastic increase from 2013 (65%) to 2022 (84%), with independent captures of n = 707 to n = 8448, respectively. Meanwhile, the percentage of photographs of domestic animals declined from 2013 (24%) to 2022 (14%), and the independent captures were observed to have increased from n = 421 to n = 2716 in the following years. The details of the percentage of camera trap photographs and independent captures from 2009 to 2022 are presented in Appendix A and Appendix B.

### 3.2. Relative Abundance Index (RAI)

In PNP, RAI analysis showed the highest tiger abundance in 2022 (RAI = 7.11) and the lowest in 2013 (RAI = 0.32), with an overall upward trend. Among the prey species, sambar’s abundance was found to increase from 2009 (RAI = 3.63) to 2018 (RAI = 8.15) but declined in 2022 (RAI = 4.96). Spotted deer’s abundance showed a rising trend from 2009 (RAI = 1.59) to 2022 (RAI = 6.57), peaking in 2018 (RAI = 10.15). Wild boar’s abundance showed fluctuation, peaking in 2013 (RAI = 12.95). Barking deer’s abundance remained relatively stable (RAI ~8), with a noticeable increase in 2018 (RAI = 11.48). Gaur exhibited relatively the lowest level of abundance across all the years, which remained at around RAI ~ 1 after 2009. Humans’ abundance dropped sharply from 2009 (RAI = 126.8) to 2022 (RAI = 14.11). Similarly, domestic animals’ abundance also declined considerably, peaking in 2013 (RAI = 54.71) and reaching the lowest value in 2022 (RAI = 1.82).

In buffer zone forests, tigers were only detected in 2022, with an RAI of 2.17. The sambar population declined from 2013 (RAI = 5.22) to 2022 (RAI = 1.06). Both spotted deer and barking deer populations showed a fluctuating trend, with the highest abundance in 2013 and the lowest in 2018. Wild boar’s abundance decreased from 2013 (RAI = 9.61) to 2022 (RAI = 1.56). On the contrary, gaur abundance showed a slight increase, from none to an RAI of 1.14 in 2022. Overall, the RAI of prey species in the buffer zone decreased over time. Interestingly, the abundance of humans and domestic animals also showed a declining trend. Humans’ abundance peaked in 2013 (RAI =395.5) and dropped by 2022 (RAI = 154.08). Likewise, domestic animals’ abundance was highest in 2013 (RAI = 164.83) and lowest in 2022 (RAI = 51.48).

In national forests, tigers were first observed in 2018 (RAI = 4.27), and their presence continued through 2022 (RAI = 2.46). Among the prey species, fluctuating trends were observed. Sambar’s abundance was found to be consistent during 2013 to 2018 (RAI~5) but declined in 2022 (RAI = 2.17). The spotted deer and barking deer populations were found to have increased by 2022, with RAIs of 5.32 and 7.98, respectively. Conversely, wild boar’s abundance exhibited a decreasing trend from 2013 (RAI = 9.14) to 2022 (RAI = 6.47). Gaur was detected from 2018 (RAI = 0.93) to 2022 (RAI = 1.14). Humans’ frequency steadily increased over the time period, rising from 2013 (RAI =351.44) to 2022 (RAI = 389.7). In contrast, domestic animals decreased from 2013 (RAI = 330.71) to 2022 (RAI = 155.02).

**Figure 4:**
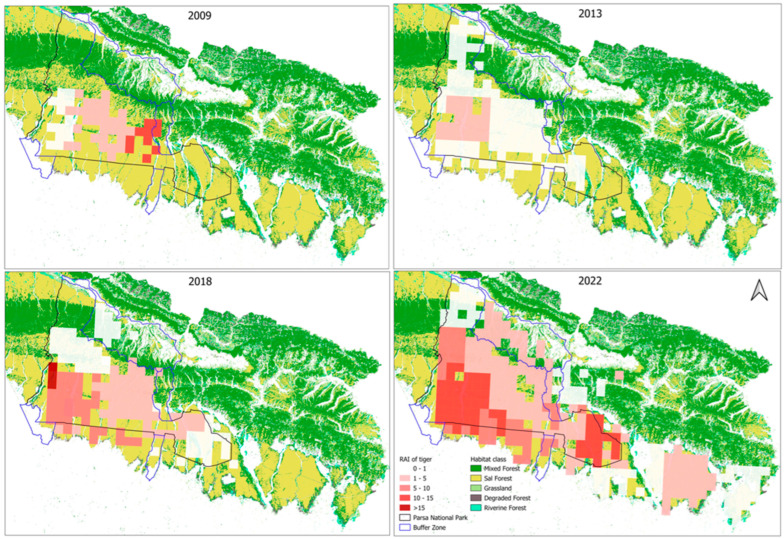
Grid-specific RAI of tigers indicated by the range of colors from white to dark red across the three forest management regimes over the time period. The grid locations beyond PNP and the buffer zone are in the national forests.

All details of the RAI values from 2009 to 2022 are given in Table 3. The visual display of the RAIs of tigers (Figure 4), prey species, and anthropogenic disturbances mapped against the habitat classifications of the TAL, as delineated by Thapa et al. [57], is provided in Supplementary Materials S2.

**Table 3:** Relative Abundance Index (RAI) values of tigers, prey, humans, and domestic animals from 2009 to 2022 across the three forest management regimes.

**Parsa National Park (PNP)**			**Year**			
**Species**	**Scientific Name**	**IUCN Status**	**2009**	**2013**	**2018**	**2022**
			**RAI (±SE)**	**RAI (±SE)**	**RAI (±SE)**	**RAI (±SE)**
Tiger	*Panthera tigris*	EN	2.65 ± 1.63	0.32 ± 0.18	5.14 ± 2.71	7.11 ± 1.58
Sambar	*Rusa unicolor*	VU	3.63 ± 1.62	7.47 ± 1.64	8.15 ± 1.05	4.96 ± 0.79
Spotted deer	*Axis axis*	LC	1.59 ± 0.76	4.11 ± 0.80	10.15 ± 6.14	6.57 ± 2.24
Wild boar	*Sus scrofa*	LC	3.07 ± 0.70	12.95 ± 2.56	4.98 ± 1.36	5.75 ± 1.01
Barking deer	*Muntiacus muntjac*	LC	7.93 ± 9.37	8.24 ± 1.05	11.48 ± 3.79	8.41 ± 1.29
Gaur	*Bos gaurus*	VU	0.20 ± 0.14	1.11 ± 0.51	0.95 ± 0.40	1.15 ± 0.40
Human	*Homo sapiens*		126.8 ± 32.70	100.07 ± 23.92	40.91 ± 11.16	14.11 ± 4.61
Domestic animals			35.25 ± 16.75	54.71 ± 19.85	8.77 ± 3.47	1.83 ± 0.77
**Buffer Zone (BZ)**						
**Species**	**Scientific Name**	**IUCN Status**		**2013**	**2018**	**2022**
				**RAI (±SE)**	**RAI (±SE)**	**RAI (±SE)**
Tiger	*Panthera tigris*	EN		0	0	2.17 ± 1.64
Sambar	*Rusa unicolor*	VU		5.22 ± 0.35	1.80 ± 0.93	1.06 ± 0.53
Spotted deer	*Axis axis*	LC		11.49 ± 10.01	1.36 ± 1.36	4.11 ± 2.39
Wild boar	*Sus scrofa*	LC		9.61 ± 7.45	4.07 ± 2.29	1.56 ± 1.28
Barking deer	*Muntiacus muntjac*	LC		11.22 ± 4.31	3.98 ± 3.98	6.96 ± 3.03
Gaur	*Bos gaurus*	VU		0	0.68 ± 0.24	1.07 ± 1.07
Human	*Homo sapiens*			395.5 ± 47.21	215.9 ± 4.99	154.08 ± 87.81
Domestic animals				164.83 ± 130.44	97.67 ± 73.12	51.48 ± 30.16
**National Forests (NFs)**						
**Species**	**Scientific Name**	**IUCN Status**		**2013**	**2018**	**2022**
				**RAI (±SE)**	**RAI (±SE)**	**RAI (±SE)**
Tiger	*Panthera tigris*	EN		0	4.27 ± 1.90	2.46 ± 1.02
Sambar	*Rusa unicolor*	VU		5.23 ± 3.41	5.8 ± 24.73	2.17 ± 1.08
Spotted deer	*Axis axis*	LC		2.54 ± 1.22	1.78 ± 0.66	5.32 ± 2.47
Wild boar	*Sus scrofa*	LC		9.14 ± 3.32	6.21 ± 1.43	6.47 ± 1.95
Barking deer	*Muntiacus muntjac*	LC		4.26 ± 2.30	3.91 ± 3.09	7.98 ± 4.31
Gaur	*Bos gaurus*	VU		0	0.93 ± 0.79	1.14 ± 0.78
Human	*Homo sapiens*			351.44 ± 221.93	364.10 ± 137.93	389.7 ± 75.00
Domestic animals				330.71 ± 281.38	248.47 ± 121.01	155.02 ± 65.98

(Note: IUCN status: EN = Endangered, VU = Vulnerable, and LC = Least Concern).

### 3.3. Test Statistics

#### 3.3.1. Hypothesis 1: There Is a Significant Difference in the Abundance of Tigers and Their Prey Species in the Three Forest Management Regimes over Time

We performed the Friedman Rank-Sum Test on the RAI values of tigers and the five prey species across all four years to determine whether there was a significant difference in the RAI values between these years. As observed from the trend, the test statistics showed a significant difference between the RAI values across the years in PNP (χ^2^ = 9.6; df = 3; *p* < 0.05), which supports our first hypothesis. In contrast, in the buffer zone (χ^2^ = 2.17; df = 2; *p* > 0.05) and national forests (χ^2^ = 1; df = 2; *p* > 0.05), there were no significant differences in the RAI values between years, which did not support the first hypothesis in those areas.

We further performed the Wilcoxon Signed-Rank Exact Test to assess pairwise differences between years in PNP. The test results showed a significant difference between the years 2009–2018 and 2009–2022 (*p* < 0.05). The details of the test values of the Friedman Rank-Sum Test, as well as of the Wilcoxon Signed-Rank Exact Test, are given in Supplementary Materials S3.

#### 3.3.2. Hypothesis 2: High Levels of Anthropogenic Disturbances from Humans and Domestic Animals Negatively Influence the Abundance of Tigers and Their Prey

**Figure 5:**
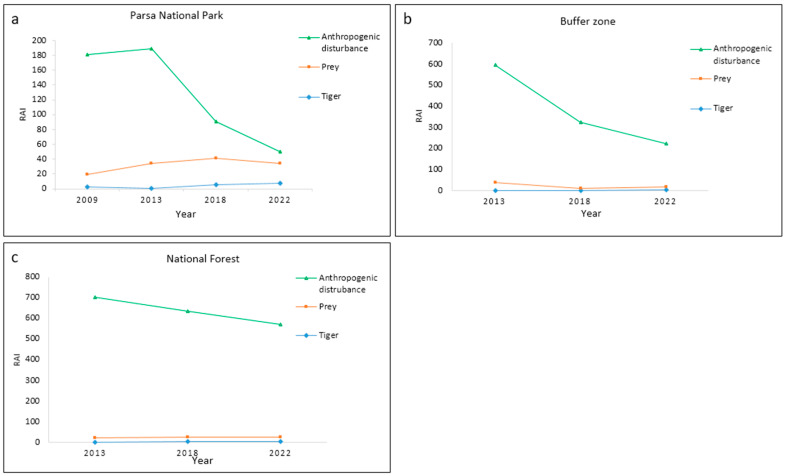
Line graphs showing abundance trends of tigers, prey, and anthropogenic disturbances from 2009 to 2022 across three forest management regimes in (**a**) Parsa National Park, (**b**) buffer zone, and (**c**) national forests.

The line graph illustrates that in PNP, the substantial reduction in anthropogenic disturbance in 2018 and 2022 led to a notable increase in the abundance of tigers, although no clear change was observed in prey species. Similarly, in the buffer zone, the gradual decrease in anthropogenic disturbances had a positive impact on the abundance of tigers but did not result in distinct changes in prey species. In national forests, a slight decrease in anthropogenic disturbances occurred over time, which could not show any distinct effect on either tigers or prey species. The details of the line graphs across three management regimes are shown in Figure 5, and the boxplots of anthropogenic disturbances are provided in Supplementary Materials S4.

**Figure 6:**
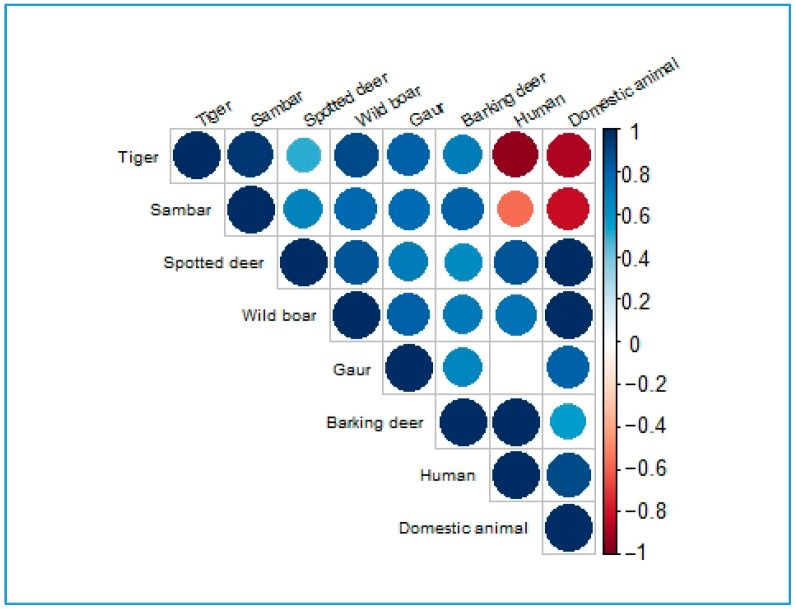
Correlation plot on the basis of the magnitudes of the coefficients. Red circles signify the intensity of negative correlation, and blue circles signify that of positive correlation.

Overall, the correlation analysis (with extreme values in parentheses) showed the following patterns: In PNP, tigers showed a positive correlation with their prey (ρ = 0.82) and a negative correlation with humans (ρ = −0.94) and domestic animals (ρ = −0.88). In the buffer zone, mainly based on 2022, tigers showed positive correlations with prey (ρ = 0.82), humans (ρ = 0.74), and domestic animals. In national forests, tigers showed a positive correlation with prey (ρ = 0.95) and a negative correlation with humans and domestic animals. Across all three forest management regimes, there were mixed relationships between prey species and both humans and domestic animals. A visual display of the correlation plot based on the magnitudes (extremes) of the coefficients is presented in Figure 6. The details of the significant correlation coefficient matrix are presented in Appendix C, and the comprehensive correlation coefficients are given in Supplementary Material S5.

The overall analysis of the line graph and correlation clearly supports the second hypothesis in PNP, while mixed results were demonstrated in the buffer zone and national forests.

## 4. Discussion

### 4.1. Percentage of Camera Trap Photographs and Independent Photo Captures

In PNP, the percentage of photos, and, consequently, the independent captures of tigers, increased over time. Among the prey species, spotted deer showed the highest increment in terms of the percentage of photos and independent captures. Conversely, gaur had the lowest and constant percentage of photos throughout the period, and the independent captures showed a slight increment. The percentage of photos and independent captures of humans and domestic animals showed a decreasing trend over time.

In the buffer zone forest, tigers were captured only in 2022. Over time, there was an increase in the number of independent captures of photos of the prey species. Although the percentage of photos of humans and domestic animals fluctuated, the independent captures for both categories showed an increase over time.

In national forests, tigers have been captured since 2018, with a consistent number of independent captures. The independent captures of prey species showed an increasing trend, except for sambar and gaur. Humans’ presence increased in percentage, and domestic animals’ presence fluctuated, but the frequency of independent captures for both rose.

### 4.2. Relative Abundance Index (RAI)

In PNP, our analysis revealed a significant increase in the number of both tigers and prey species over time, with notable changes observed after 2018, with the highest RAI in 2022 (RAI = 7.11). Although the patterns of prey species were found to slightly fluctuate, the overall RAI from 2009 to 2022 was found to be in an upward trend. Our study results align with the density of tigers and prey species in PNP from the Tiger Count Reports of Nepal, which recorded 0.72/100 sq. km in 2009 to the highest density of 1.74/100 sq. km in 2022 of tigers and the density of prey species, which rose from 5.5/sq. km in 2009 to 75.1/sq. km in 2022 [39,40,51,52]. A study conducted by Lamichhane et al. [58] also showed the increment in tiger numbers from 2013 to 2016 in PNP. However, we consistently observed the lowest RAI for gaur each year, a finding similar to the results reported by the DNPWC and DFSC [40]. Compared with Chitwan National Park (CNP), an adjoining national park, which showed much higher RAI values (i.e., the detection rate taken at 60 min intervals of independent captures of photographs per 100 trap days) of 5.692 and 7.973 for tigers and all five prey species in the years 2018 and 2022 [59]. Although PNP and CNP are adjoining parks, the lower abundance of tigers and prey species in PNP compared with CNP may be because of its location in the Bhabar region, which has dry habitat conditions and severe water scarcity [46,60].

However, the conservation interventions (Figure 2), such as village evacuation, area extension, security enhancement, and status upgradation, undertaken within the period of 2009 to 2022, were found to be effective as the abundance of tigers and their prey was found to be significantly increased over time, supporting our first hypothesis.

Compared with Ranthambore National Park (RNP) in India, which has a higher abundance of tigers (RAI = 19.45) and prey species [30], Manas National Park (MNP) reported a slightly lower abundance of tigers (RAI = 4.09) and a higher abundance of prey species [35] than PNP. Similarly, the prey abundance in the Nagarjunsagar Srisailam Tiger Reserve (NSTR) was found to be higher [31] than in PNP. In contrast, PNP showed a higher abundance of tigers and prey species compared with the Similipal Tiger Reserve (STR) and the Kuldiha Wildlife Sanctuary (KWS), and in India [33,61]. These comparisons indicate a clear positive relation between tiger abundance and prey availability.

Our findings revealed that the RAI of anthropogenic disturbances drastically decreased from 2009 (RAI = 162.19) to 2022 (RAI = 15.91) in PNP. This indicates the strengthening of the park security system as well as a conducive environment for the wildlife, supporting our second hypothesis. However, nominal trespassers need to be controlled in the future. Compared with RNP, where there were low levels of disturbances (domestic animals: RAI ~ 6), the abundance of tigers was found to be higher [30]. Conversely, STR, where there were higher levels of anthropogenic disturbances (RAI > 20), showed a lower abundance of tigers (RAI = 0.02) [61]. Likewise, in comparison with KWS and NSTR [31,33], PNP experienced lower levels of anthropogenic disturbances.

In the buffer zone forest, we observed no significant difference in the RAI values of tigers and prey species over time (χ^2^ = 2.17; df = 2; *p* > 0.05). However, the detection of tigers in 2022 indicated a clear spillover population of tigers from PNP. A study on the ecological carrying capacity showed that PNP can hold a maximum of 39 tigers in the park [41]. Hence, the buffer zone forest served as a good refuge for the spill-over tiger population. Meanwhile, the declining trend in the abundance of the four major prey species was found to be a serious issue in the buffer zone, as it could exacerbate human–tiger conflicts. Although gaur’s abundance was found to be nominally increased, it was limited only to the foothills of the Churiya range. Surprisingly, our results showed a decreasing trend in anthropogenic disturbances, as well as in the buffer zone forest. Such a result may be due to the effective conservation education programs in the buffer zone villages, where 50% of the park revenue goes back for their development and conservation activities. Similarly, a decreasing trend in anthropogenic disturbances was also observed in the buffer zone of CNP, with decreased reliance on agriculture and livestock due to foreign employment of youths and stall-feeding practices [62]. Dhungana et al. [63] and Silwal et al. [64] found that the majority of human–tiger conflicts occurred during fodder or fuelwood collection in the buffer zone forest of CNP. Similarly, a study conducted in the Panna Tiger Reserve, India, showed that domestic animals were the majority of prey animals killed by tigers in the multiple-use buffer zone [65]. Although the reduction in anthropogenic pressure is a positive sign for wildlife conservation, the decreasing trend in the presence of prey species and the increasing presence of tigers in the buffer zone forest might cause an increase in human–tiger conflicts in the future, and it needs to be addressed in a timely manner.

In the national forests, there has been no significant difference in the RAI values of tigers and prey species over time (χ^2^ = 1; df = 2; *p* > 0.05). However, the capture of tigers since 2018 indicated a continuous presence and favorable habitat of tigers in the forests. Meanwhile, a fluctuating trend in the abundance of prey species was shown by our results in these forests. Such fluctuation in the abundance of prey species tends to occur in community-owned forests due to shortages of food and water in dry periods [66]. In contrast, human pressure remained consistently high and is rising in the area, posing a serious challenge for tiger conservation and exacerbating human–tiger conflicts. As the studies by Gurung et al. [67] and Acharya et al. [66] highlighted, tiger attacks on humans primarily occurred within forests. In comparison, the Ramnagar Forest Division (outside the protected area) of North India contained a higher abundance of tigers (RAI = 6.03) and a higher abundance of prey species and had a lower level of anthropogenic disturbance [36] than these national forests. Interestingly, the decrease in the RAI of domestic animals over time may be a positive sign for ungulate conservation. This trend for domestic animals clearly indicates that local households are reducing their livestock, even in the peripheral villages of national forests. However, some research has indicated that livestock constitutes a significant portion of tigers’ diet in multiple-use forests, which leads to conflict hotspots [66]. Hence, a contrasting scenario has been revealed in national forests, which could lead to more human–tiger conflicts in the future. In response, implementing coexistence strategies, such as conflict mapping, improved livestock husbandry, regular tiger monitoring, prey base enhancement, awareness campaigns, and reducing human pressure during the peak time when tigers are active, could be effective in mitigating future conflicts [68,69].

In all three forest management regimes, there has been a noticeable decrease in domestic animals, clearly indicating that local villagers are reducing them. A similar trend was reported by Lamichhane et al. [62] in CNP, an adjoining park. Although the research identified some of the reasons, such as off-farm-based income, like foreign employment, stall feeding practices were the cause of the reduction in livestock in forests, as well as per household. We recommend further study for an in-depth understanding of the root cause and the potential impacts of this.

### 4.3. Correlation

Overall, our findings indicated that tigers were positively correlated with the prey species, which is consistent with the results of Yang et al. [70]. Similarly, previous studies have shown that tiger density was positively correlated with prey density, as reported by O’Brien et al. [43], Karanth et al. [5], and Hayward et al. [71]. These correlations highlight the ecological dependency of tigers on prey availability for their survival. In PNP and national forests, tigers exhibited a negative correlation with both humans and domestic animals, aligning with the findings of Yang et al. [70] and Acharya et al. [66], suggesting that tigers tend to avoid areas with human disturbances. However, in the buffer zone, tigers showed a positive correlation with humans and domestic animals, a pattern that requires further investigation using comprehensive multiple years of datasets. A similar positive correlation between tigers and domestic animals at the park boundary of Chitwan National Park (CNP) was reported by Dhungana et al. [68]. Likewise, studies from the peripheries of protected areas in India and China have shown that areas with higher human activity and livestock grazing were associated with reduced wild ungulate abundance, which, in turn, causes tigers to prey on livestock [14,72]. Together, these findings underscore the complex interactions between tigers, prey, humans, and domestic animals, particularly in human-dominated landscapes.

Although prey showed mixed relationships with humans and domestic animals, we also observed that spotted deer mostly exhibited a positive correlation with both, which is likely due to their tendency to avoid predators and seek safer environments. Several studies, such as those by Muhly et al. [73] and Lamichhane et al. [74], showed a higher positive association between prey species and humans than predators, supporting the human shield hypothesis. In contrast, other prey species displayed mixed relationships with humans and domestic animals. However, studies conducted in CNP and the Kuldiha Wildlife Sanctuary (KWS), India, have reported that major prey species had a negative association with human disturbances [33,75].

## 5. Policy Implications, Limitations, and Future Research

This study highlights the importance of tailored forest management regimes in supporting tiger populations and their prey. The success of interventions in Parsa National Park (PNP), such as village evacuation, park expansion, and enhanced security, demonstrates the effectiveness of strict protection and habitat restoration. Policymakers should consider applying similar measures in comparable protected areas to boost tiger recovery.

The presence of tigers in buffer zones and national forests despite fluctuating prey and rising human activity calls for adaptive policies beyond strict protection. Strengthening prey management, community engagement, and conflict mitigation in buffer zones is essential to prevent human–tiger conflicts. Future research should prioritize in-depth investigations into the dynamics of human–tiger interactions in buffer zones and national forests to inform timely and effective conflict mitigation strategies. The decline in domestic animals across all regimes suggests shifts in rural livelihoods and grazing patterns. Encouraging sustainable livelihood options and improved livestock management can reduce forest dependency and conflict risks. Further socio-economic research is needed to guide these interventions. Also, understanding the ecological interactions between sympatric carnivores in the area will further enrich our knowledge base for conservation planning.

Limitations include the data collection being restricted to winter–spring seasons, excluding seasonal variations in wildlife and human activity. Future research should implement year-round monitoring and incorporate factors like deforestation, poaching, and socio-economic drivers for a comprehensive understanding. The RAI used has inherent biases, including imperfect detection, variation in detectability across sites and species, and the influence of animal movement behavior [27,28]. Integrating the RAI with advanced modeling techniques, such as occupancy or spatially explicit capture–recapture models, can help improve the accuracy of population estimates. Similarly, the relatively lower number of camera trap points in the buffer zone may affect the robustness of the analysis in that area.

Finally, assessing the ecological connectivity of the Parsa–Chitwan–Valmiki landscape through collaborative transboundary research is vital for coordinated regional conservation efforts, especially against poaching and habitat fragmentation.

## 6. Conclusions

This long-term study (2009–2022) offers a valuable insight into tiger populations, prey dynamics, and anthropogenic pressures across three forest management regimes. In PNP, conservation efforts such as habitat expansion and enhanced protection have driven notable increases in the abundance of both tigers and prey, while also curbing the illegal entry of humans and domestic animals. In contrast, the buffer zone, despite a growing tiger presence, showed a concerning decline in prey abundance. This highlights the urgent need for targeted prey recovery and enhanced community-based management. In national forests, the consistent presence of tigers since 2018 signals untapped potential for conservation expansion. However, fluctuating prey numbers and persistent human disturbances remain significant challenges. The observed patterns in national forests indicated that with low prey availability and high human disturbances, there may be a potential risk of an increase in human–tiger conflicts. However, the steady decline in domestic animals across all three forest management regimes indicates a promising shift that could help reduce pressure on the forests.

Taken together, these findings reinforce that tiger conservation success depends on an integrated approach that combines effective forest management practice, sufficient prey availability, and effective control of anthropogenic disturbances across a connected landscape. By addressing ongoing threats and building on existing gains, there is a clear opportunity to expand tiger conservation beyond protected areas and ensure the long-term survival of tigers across this transboundary landscape.

## Figures and Tables

**Table 1:** Summary of the study area.

	Parsa National Park	Buffer Zone	National Forests
Area (Sq. km)	627.39	285.3	845.25
Conservation status	Fully protected; restricted for people	Less-restricted forest; locals can utilize forest products	Least-restricted forest; locals and government utilize and sell forest products
Population	-	~85,000	~2,000,000
Precipitation	1200–2500 mm		
Temperature (range)	7–39 °C		
Elevation (meter)	100–950		
Climatic zone	Sub-tropical		

Source: [46,47].

**Table 2:** Number of camera trap grids in three different forest management regimes in different years.

Year	Parsa National Park	Buffer Zone	National Forests	Total Camera Trap Grids
2009	117			117
2013	136	11	30	177
2018	219	30	56	305
2022	201	37	126	364

(Note: The same camera trap grids were consistently used across all survey years. In each successive year, additional grids were added based on new evidence of tiger presence (sources: [39,40,51,52]).

## Data Availability

Data can be provided upon a genuine request to the first author.

## Supplementary Materials

The following supporting information can be downloaded at https://www.mdpi.com/article/10.3390/ani15182697/s1, Table S1: Details of the camera traps across the three forest management regimes from 2009 to 2022, Figure S1a: Grid specific RAI of prey species indicated by range of colors from white to dark red across the three forest management regimes over the time period. Prey species refers to sambar, spotted deer, wild boar, barking deer and gaur all together. The grid locations beyond PNP and buffer zone are of the national forests, Figure S1b: Grid specific RAI of anthropogenic disturbance indicated by range of colors from white to dark red across the three forest management regimes over the time period. The grid locations beyond PNP and buffer zone are of the national forests, Table S2: Friedman Rank Sum Test in R for tigers and five prey species of RAI between four years, Figure S2: Boxplots showing the anthropogenic disturbances across three forest management regimes from 2009 to 2022, S5 (correlation): Spearman correlation coefficients.

## Appendix B. The Numbers of Independent Photos Captured for Tigers, Prey Species, Humans, and Domestic Animals from 2009 to 2022

**Parsa National Park (PNP)**				
**Species**	**2009**	**2013**	**2018**	**2022**
Tiger	53	11	125	201
Sambar	52	206	171	148
Spotted deer	19	101	325	219
Wild boar	52	334	160	176
Barking deer	143	202	329	239
Gaur	4	34	28	37
Human	1822	2307	1472	449
Domestic animals	407	1131	288	63
**Buffer Zone (BZ)**				
**Species**		**2013**	**2018**	**2022**
Tiger		0	0	38
Sambar		9	5	12
Spotted deer		24	3	38
Wild boar		20	11	26
Barking deer		22	18	94
Gaur		0	2	16
Human		705	731	3376
Domestic animals		334	413	1054
**National Forests (NFs)**				
**Species**		**2013**	**2018**	**2022**
Tiger		0	44	40
Sambar		14	60	24
Spotted deer		11	8	77
Wild boar		28	31	100
Barking deer		13	7	79
Gaur		0	43	23
Human		707	1961	8448
Domestic animals		421	1047	2716

## Appendix C. Spearman Correlation Coefficient Matrix (0.5≥) of Relative Abundance Index (RAI) of Tiger, Prey Species, and Anthropogenic Disturbance

	**Tiger**	**Sambar**	**Spotted Deer**	**Wild Boar**	**Gaur**	**Barking Deer**	**Human**	**Domestic Animals**
**Tiger**	1							
**Sambar**	0.70 ^2a^	1						
	0.63 ^4b^	1						
	0.95 ^3c^	1						
**Spotted deer**	0.50 ^4c^	0.67 ^2a^	1					
**Wild boar**	0.82 ^1a^	0.55 ^2a^	0.85 ^3a^	1				
	0.89 ^4c^	0.78 ^4c^	0.75 ^4c^	1				
**Gaur**	0.55 ^3a^	0.60 ^3a^	0.70 ^2a^	0.68 ^3a^	1			
	0.82 ^4b^			0.82 ^4b^	1			
	0.65 ^3c^	0.77 ^3c^		−0.50 ^3c^	1			
**Barking deer**	0.52 ^1a^	0.52 ^3a^		0.63 ^3a^		1		
			0.63^4b^			1		
	0.70 ^3c^	0.82 ^4c^		0.71 ^4c^	0.66 ^3c^	1		
				−0.65 ^3c^		1		
**Human**	−0.94 ^1a^		0.85 ^3a^	0.65 ^3a^		−0.66 ^1a^	1	
				−0.71 ^1a^			1	
	0.74 ^4b^	0.5 ^2b^	0.5 ^2b^	0.74 ^4b^		1 ^2b^	1	
		−0.56 ^2c^	0.66 ^3c^	0.61 ^3c^			1	
**Domestic animals**	−0.88 ^1a^		0.93 ^4a^	0.71 ^3a^	−0.57 ^3a^	0.57 ^3a^	0.89 ^3a^	1
			0.73 ^3a^	−0.54 ^1a^		−0.54 ^1a^		1
		0.63 ^4b^	1 ^2b^	1 ^4b^	0.82 ^4b^	0.5 ^2b^	0.74 ^4b^	1
		−0.82 ^2c^	0.77 ^3c^	0.66 ^3c^				1
Note: Years: 2009 ^1^, 2013 ^2^, 2018 ^3^, and 2022 ^4^. Management regimes: PNP ^a^, buffer zone ^b^, and national forests ^c^.								
