# Supplementary material for: Revival of Tigers: Long-Term Trends (2009–2022) in the Relative Abundance Index of Tigers, Prey, and Anthropogenic Disturbance in Parsa National Park, Nepal
Source: Animals (Basel). 2025 Sep 15;15(18):2697. doi: 10.3390/ani15182697 (PMC12466492; doi:10.3390/ani15182697)
Supplement: Supplementary file 1 [file animals-15-02697-s001.zip › animals-3847491-supplementary.pdf]

Supplementary Material- S1 (Table of camera traps)

Table S1: Details of the camera traps across the three forest management regimes from 2009 to 2022

**Year**	**Location**	**Camera trap points**	**Total days**	**Mean days**	**Standard deviation (±days)**	**Range (days)**
2009	PNP	105	1579	15.04	2.6	4-18
2013	PNP	132	2268	17.18	2.05	9-22
	BZ	10	173	17.00	0.95	16-19
	NF	27	483	17.89	1.28	16-20
2018	PNP	171	2871	16.79	2.79	5-21
	BZ	20	336	16.80	3.29	8-20
	NF	35	605	17.29	2.53	5-20
2022	PNP	158	2834	17.94	4.73	4-36
	BZ	36	606	16.83	6.65	3-36
	NF	125	2098	16.78	3.66	4-36

Supplementary Material- S2 (maps)

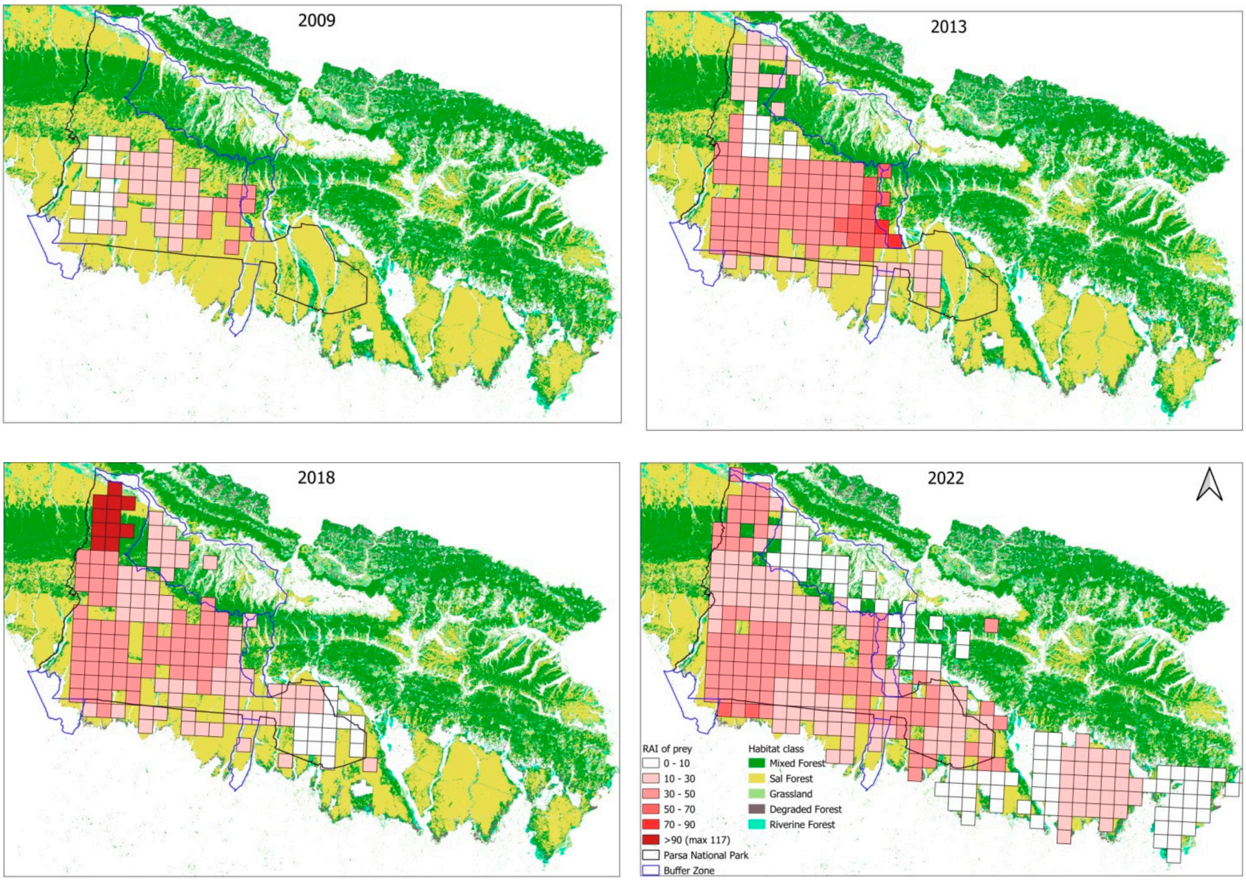

Figure S1a: Grid specific RAI of prey species indicated by range of colors from white to dark red across the three forest management regimes over the time period. Prey species refers to sambar, spotted deer, wild boar, barking deer and gaur all together. The grid locations beyond PNP and buffer zone are of the national forests.

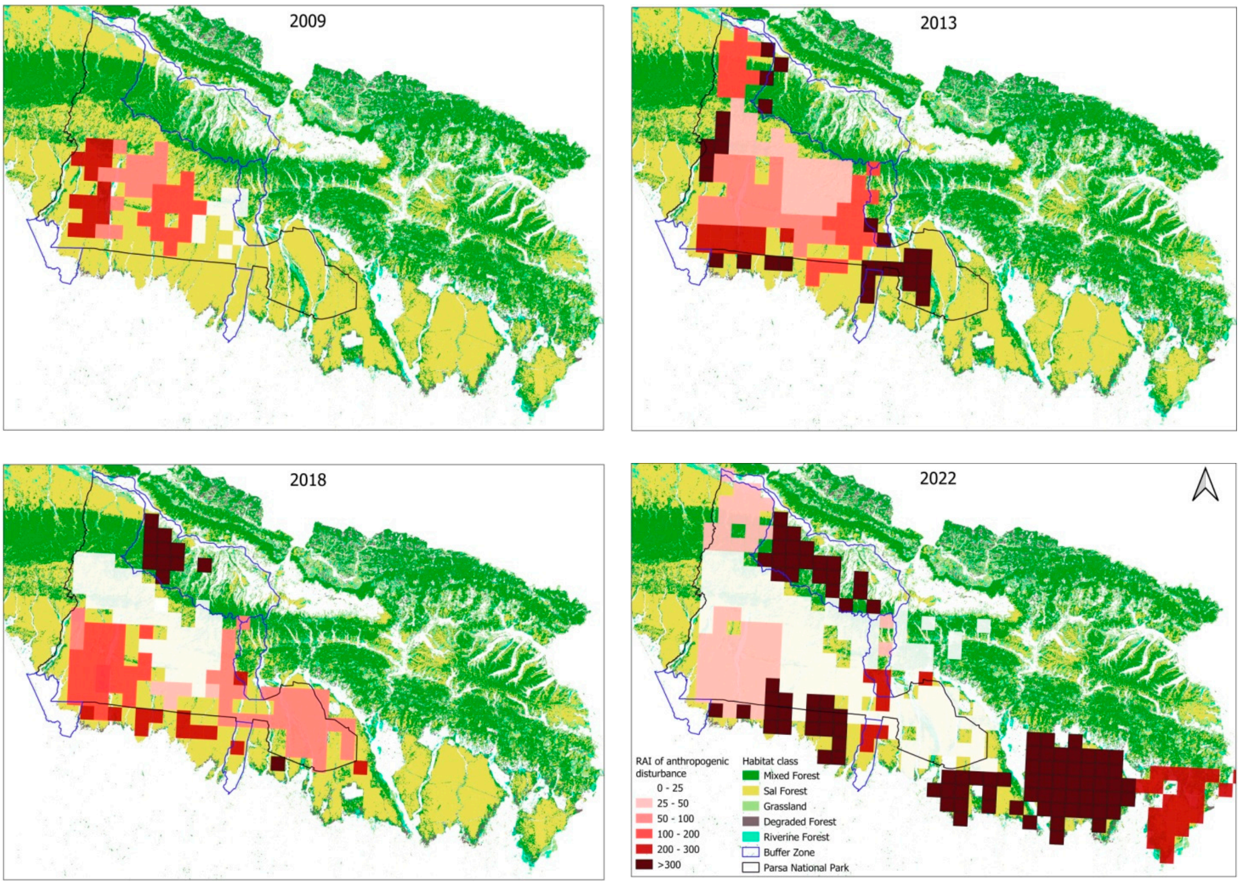

Figure S1b: Grid specific RAI of anthropogenic disturbance indicated by range of colors from white to dark red across the three forest management regimes over the time period. The grid locations beyond PNP and buffer zone are of the national forests.

Supplementary material- S3 (Friedman and Wilcoxon)

Table S2: Friedman Rank Sum Test in R for tigers and 5 prey species of RAI between four years

**Friedman test**			
**Location**	**χ^2 value**	**df**	**p-value**
**PNP**	**9.6**	**3**	**0.02229**
BZ	2.1739	2	0.3372
NF	1	2	0.6065

**Wilcoxon test (Post-hoc test)**		
**Years (paired)**	**W_{stat}**	**p-value**
2009-2013	3	0.1563
**2009-2018**	**0**	**0.03125**
**2009-2022**	**0**	**0.03125**
2013-2018	7	0.5625
2013-2022	10	1.00
2018-2022	6	0.4375

Supplementary Material- S4 (anthropogenic boxplot)

Figure S2: Boxplots showing the anthropogenic disturbances across three forest management regimes from 2009 to 2022.

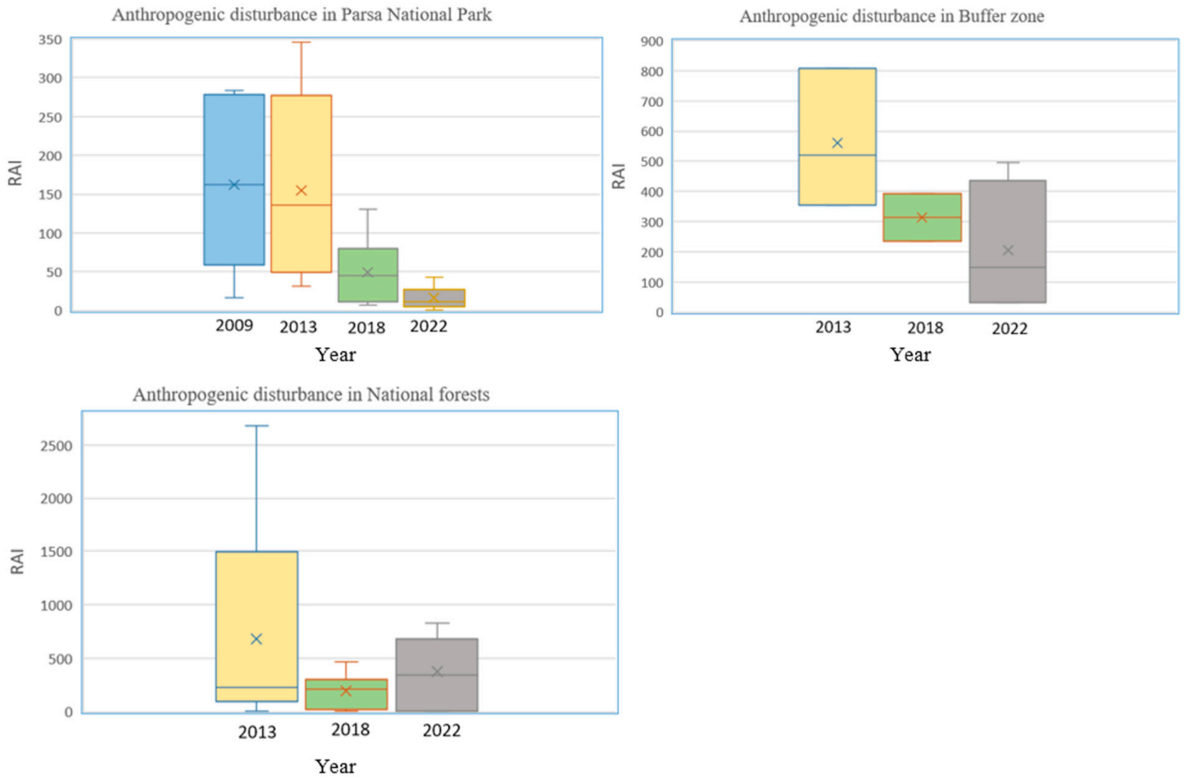

Anthropogenic disturbances (combine RAI of humans and domestic animals) using boxplots measure displayed the highest reduction in RAI of anthropogenic disturbances from 2009 (*Mean*=162.19, *Median*=162.04) to 2022 (*Mean*=15.91, *Median*=10.65) in PNP. Similarly, at the buffer zone, a notable reduction was observed from 2013 (*Mean*=560.30, *Median*=519.7) to 2022 (*Mean*=205.56, *Median*=147.83). In national forests, there was only slight reduction from 2013 (*Mean*=682.14, *Median*=227.02) to 2022 (*Mean*=373.19, *Median*=345.98). Such slight reduction in national forests was also attributed only by the decrease of domestic animals.

Supplementary material S5 (correlation)

**Spearman correlation coefficent-2022 (PNP)**								
	RAI.tiger	RAI.sambar	RAI.spotted.deer	RAI.wildboar	RAI.barking.deer	RAI.Gaur	RAI.Human	RAI.Domestic.animal
RAI.tiger	1	-0.1190476	0.285714286	0.47619048	-0.682646969	-0.42857	-0.0714286	0.121987509
RAI.sambar	-0.11905	1	-0.476190476	0.11904762	0.45509798	-0.11905	-0.1904762	-0.317167524
RAI.spotted.deer	0.28571	-0.4761905	1	0.40476191	-0.526955555	-0.07143	0.452381	0.927105069
RAI.wildboar	0.47619	0.11904762	0.404761905	1	-0.239525252	0.238095	0.1666667	0.195180015
RAI.barking.deer	-0.68265	0.45509798	-0.526955555	-0.2395253	1	0.323359	-0.1676677	-0.343616486
RAI.Gaur	-0.42857	-0.1190476	-0.071428571	0.23809524	0.323359091	1	0.452381	-0.195180015
RAI.Human	-0.07143	-0.1904762	0.452380952	0.16666667	-0.167667677	0.452381	1	0.292770022
RAI.Domestic.animal	0.12199	-0.3171675	0.927105069	0.19518002	-0.343616486	-0.19518	0.29277	1
**Spearman correlation coefficent-2022 (BZ)**								
	RAI.tiger	RAI.sambar	RAI.spotted.deer	RAI.wildboar	RAI.barking.deer	RAI.Gaur	RAI.Human	RAI.Domestic.animal
RAI.tiger	1	0.63245553	-0.055555556	1	0.816496581	-0.10541	0.7378648	1
RAI.sambar	0.63246	1	0.737864787	0.63245553	0.774596669	0.4	0	0.632455532
RAI.spotted.deer	-0.05556	0.73786479	1	-0.0555556	0.272165527	0.632456	-0.6324555	-0.055555556
RAI.wildboar	1	0.63245553	-0.055555556	1	0.816496581	-0.10541	0.7378648	1
RAI.barking.deer	0.8165	0.77459667	0.272165527	0.81649658	1	-0.2582	0.2581989	0.816496581
RAI.Gaur	-0.10541	0.4	0.632455532	-0.1054093	-0.25819889	1	-0.2	-0.105409255
RAI.Human	0.73786	0	-0.632455532	0.73786479	0.25819889	-0.2	1	0.737864787
RAI.Domestic.animal	1	0.63245553	-0.055555556	1	0.816496581	-0.10541	0.7378648	1
**Spearman correlation coefficent-2022 (NF)**								
	RAI.tiger	RAI.sambar	RAI.spotted.deer	RAI.wildboar	RAI.barking.deer	RAI.Gaur	RAI.Human	RAI.Domestic.animal
RAI.tiger	1	0.70418685	0.5	0.89285714	0.453163484	0.678571	0.0714286	-0.357142857
RAI.sambar	0.70419	1	0.333562192	0.77831178	0.306697808	0.815374	-0.1853123	-0.148249863
RAI.spotted.deer	0.5	0.33356219	1	0.75	0.157622081	0.214286	0.25	0.428571429
RAI.wildboar	0.89286	0.77831178	0.75	1	0.453163484	0.714286	0	-0.071428571
RAI.barking.deer	0.45316	0.30669781	0.157622081	0.45316348	1	0.394055	-0.3743524	-0.01970276
RAI.Gaur	0.67857	0.81537425	0.214285714	0.71428571	0.394055203	1	0.0714286	-0.178571429
RAI.Human	0.07143	-0.1853123	0.25	0	-0.374352443	0.071429	1	0.392857143
RAI.Domestic.animal	-0.35714	-0.1482499	0.428571429	-0.0714286	-0.01970276	-0.17857	0.3928571	1

**Spearman correlation coefficent- 2018 (PNP)**								
	RAI.tiger	RAI.sambar	RAI.spotted.deer	RAI.wildboar	RAI.Gaur	RAI.barking.deer	RAI.Human	RAI.Domestic.animal
RAI.tiger	1	0.28333333	0.376572334	0.36666667	-0.034815531	0.433333333	0.4166667	0.452601905
RAI.sambar	0.283333	1	0.092051015	0.31666667	-0.078334945	0.516666667	0.0666667	0.104446594
RAI.spotted.deer	0.376572	0.09205102	1	0.84519568	-0.47198037	0.284521319	0.8451957	0.734191687
RAI.wildboar	0.366667	0.31666667	0.845195683	1	-0.678902858	0.283333333	0.65	0.705014507
RAI.Gaur	-0.034816	-0.07833495	-0.47198037	-0.6789029	1	0.200189304	-0.356859	-0.572727273
RAI.barking.deer	0.433333	0.51666667	0.284521319	0.28333333	0.200189304	1	-0.083333	-0.147966008
RAI.Human	0.416667	0.06666667	0.845195683	0.65	-0.356859195	-0.083333333	1	0.887796045
RAI.Domestic.animal	0.452602	0.10444659	0.734191687	0.70501451	-0.572727273	-0.147966008	0.887796	1

**Spearman correlation coefficent- 2018 (NF)**								
	RAI.tiger	RAI.sambar	RAI.spotted.deer	RAI.wildboar	RAI.barking.deer	RAI.Gaur	RAI.Human	RAI.Domestic.animal
RAI.tiger	1	0.95486371	-0.333947207	-0.3946649	0.695608344	0.646843159	-0.030359	-0.212511859
RAI.sambar	0.954864	1	-0.231908414	-0.3478626	0.664211164	0.632352941	0.2029199	-0.115954207
RAI.spotted.deer	-0.333947	-0.23190841	1	0.6	-0.392792202	0.202919862	0.6571429	0.771428571
RAI.wildboar	-0.394665	-0.34786262	0.6	1	-0.654653671	-0.49280538	0.4857143	0.371428571
RAI.barking.deer	0.695608	0.66421116	-0.392792202	-0.6546537	1	0.664211164	-0.392792	0.130930734
RAI.Gaur	0.646843	0.63235294	0.202919862	-0.4928054	0.664211164	1	0.0579771	0.31887407
RAI.Human	-0.030359	0.20291986	0.657142857	0.48571429	-0.392792202	0.057977104	1	0.314285714
RAI.Domestic.animal	-0.212512	-0.11595421	0.771428571	0.37142857	0.130930734	0.31887407	0.3142857	1

**Spearman correlation coefficent- 2013 (PNP)**								
	RAI.tiger	RAI.sambar	RAI.spotted.deer	RAI.wildboar	RAI.barking.deer	RAI.Gaur	RAI.Human	RAI.Domestic.animal
RAI.tiger	1	0.7030048	0.297044263	0.60399	0.119315895	0.55389	-0.2970443	-0.425763443
RAI.sambar	0.7030048	1	0.666666667	0.55	0.234311674	0.54245	0.0666667	0.033333333
RAI.spotted.deer	0.2970443	0.6666667	1	0.53333333	0.175733756	0.69502	0.4	0.2
RAI.wildboar	0.60399	0.55	0.533333333	1	0.443518527	0.5255	0.2333333	-0.033333333
RAI.barking.deer	0.1193159	0.2343117	0.175733756	0.44351853	1	0.49366	-0.1255241	-0.083682741
RAI.Gaur	0.5538909	0.5424508	0.695015091	0.52549922	0.493657248	1	-0.0678064	-0.169515876
RAI.Human	-0.297044	0.0666667	0.4	0.23333333	-0.125524111	-0.0678	1	0.85
RAI.Domestic.animal	-0.425763	0.0333333	0.2	-0.03333333	-0.083682741	-0.1695	0.85	1
**Spearman correlation coefficent- 2013 (BZ)**								
	RAI.tiger	RAI.sambar	RAI.spotted.deer	RAI.wildboar	RAI.barking.deer	RAI.Gaur	RAI.Human	RAI.Domestic.animal
RAI.tiger	1	NA	NA	NA	NA	NA	NA	NA
RAI.sambar	NA	1	0.5	0.5	-0.5	NA	-0.5	0.5
RAI.spotted.deer	NA	0.5	1	1	0.5	NA	0.5	1
RAI.wildboar	NA	0.5	1	1	0.5	NA	0.5	1
RAI.barking.deer	NA	-0.5	0.5	0.5	1	NA	1	0.5
RAI.Gaur	NA	NA	NA	NA	NA	1	NA	NA
RAI.Human	NA	-0.5	0.5	0.5	1	NA	1	0.5
RAI.Domestic.animal	NA	0.5	1	1	0.5	NA	0.5	1
**Spearman correlation coefficent- 2013 (NF)**								
	RAI.tiger	RAI.sambar	RAI.spotted.deer	RAI.wildboar	RAI.barking.deer	RAI.Gaur	RAI.Human	RAI.Domestic.animal
RAI.tiger	1	0.7030048	0.297044263	0.60399	0.119315895	0.55389	-0.2970443	-0.425763443
RAI.sambar	0.7030048	1	0.666666667	0.55	0.234311674	0.54245	0.0666667	0.033333333
RAI.spotted.deer	0.2970443	0.6666667	1	0.53333333	0.175733756	0.69502	0.4	0.2
RAI.wildboar	0.60399	0.55	0.533333333	1	0.443518527	0.5255	0.2333333	-0.033333333
RAI.barking.deer	0.1193159	0.2343117	0.175733756	0.44351853	1	0.49366	-0.1255241	-0.083682741
RAI.Gaur	0.5538909	0.5424508	0.695015091	0.52549922	0.493657248	1	-0.0678064	-0.169515876
RAI.Human	-0.297044	0.0666667	0.4	0.23333333	-0.125524111	-0.0678	1	0.85
RAI.Domestic.animal	-0.425763	0.0333333	0.2	-0.03333333	-0.083682741	-0.1695	0.85	1

**Spearman correlation coefficent- 2009 (PNP)**								
	RAI.tiger	RAI.sambar	RAI.spotted.deer	RAI.wildboar	RAI.Gaur	RAI.barking.deer	RAI.Human	RAI.Domestic.animal
RAI.tiger	1	0.2125119	0.154010276	0.8196886	0.3592106	0.51610023	-0.941124	-0.880406274
RAI.sambar	0.2125	1	-0.202919862	0.6	-0.3718679	-0.085714286	-0.142857	0.085714286
RAI.spotted.deer	0.154	-0.2029199	1	0.0579771	-0.2229482	0.144942759	-0.40584	0.144942759
RAI.wildboar	0.8197	0.6	0.057977104	1	0.3380617	0.314285714	-0.714286	-0.542857143
RAI.Gaur	0.3592	-0.3718679	-0.222948161	0.3380617	1	0.169030851	-0.169031	-0.507092553
RAI.barking.deer	0.5161	-0.0857143	0.144942759	0.31428571	0.1690309	1	-0.657143	-0.542857143
RAI.Human	-0.9411	-0.1428571	-0.405839725	-0.71428571	-0.1690309	-0.657142857	1	0.771428571
RAI.Domestic.animal	-0.8804	0.0857143	0.144942759	-0.54285714	-0.5070926	-0.542857143	0.7714286	1
